# Safe Fall: Use of Predictive Modeling and Machine Vision Techniques for Fall Analysis and Fall Quality

**DOI:** 10.3390/s26082491

**Published:** 2026-04-17

**Authors:** O. DelCastillo-Andrés, R. Fernández-García, J. C. Pastor-Vicedo, M. A. Lira, M. C. Campos-Mesa, C. Castañeda-Vázquez, E. Genovesi, S. Krstulović, G. Kuvačić, K. Morvay-Sey, R. Sánchez-Reolid

**Affiliations:** 1 Department of Physical Education and Sport, Area of Didactics of Body Expression, Faculty of Educational Sciences, University of Seville, 41009 Seville, Spain; mccampos@us.es (M.C.C.-M.); carolinacv@us.es (C.C.-V.); 2 Department of Electronic Engineering, Universitat Politècnica de Catalunya, 08222 Terrassa, Spain; raul.fernandez-garcia@upc.edu; 3 Facultad de Educación de Albacete, Universidad de Castilla-La Mancha, 02071 Albacete, Spain; juancarlos.pastor@uclm.es; 4 Department of Applied Didactics, Physical Education Section, Faculty of Education, University of Barcelona (UB), 08007 Barcelona, Spain; mlira@ub.edu; 5 USchool of Exercise and Sport Sciences, Department of Biomedical Sciences for Health, Università degli Studi di Milano, 20122 Milan, Italy; edoardo.genovesi@unimi.it; 6 Faculty of Kinesiology, University of Split, 21000 Split, Croatia; sasa@kifst.hr (S.K.); goran.kuvacic@kifst.eu (G.K.); 7 Institute of Physiotherapy and Sport Sciences, Faculty of Health Sciences, University of Pécs, 7621 Pécs, Hungary; kata.sey@etk.pte.hu; 8 Department of Information Technologies and Systems, Faculty of Social Sciences and Information Technologies, University of Castilla-La Mancha, 45600 Talavera de la Reina, Spain

**Keywords:** fall detection, safe falling, protective strategies, computer vision, SAM 2, machine learning, injury prevention, biomechanics

## Abstract

Falls are a leading cause of paediatric injuries, yet school-based prevention relies heavily on subjective observation rather than objective biomechanical assessment. This paper introduces the *Safe Fall* framework, integrating a judo-inspired educational programme with an occlusion-robust computer vision pipeline to quantify safe falling strategies. We analysed video recordings of 285 schoolchildren using a multi-stage architecture combining YOLOv8 for detection, SAM 2 for segmentation, and MMPose for skeletal tracking. The intervention yielded significant improvements in 60% of kinematic metrics (p<0.05), most notably a +61.4% increase in descent rate and expanded rolling ranges, indicating a shift from hazardous “freezing” behaviours to controlled energy dissipation. Unsupervised clustering confirmed a migration of students towards safe motor profiles, while a Random Forest classifier achieved an accuracy of 98.3% and an AUC of 0.998 in distinguishing fall quality. These findings demonstrate that integrating pedagogical training with automated vision modelling provides a scalable and evidence-based approach for reducing injury risk in real-world school environments.

## 1. Introduction

Falls constitute a pervasive public health crisis, identified by the World Health Organisation (WHO) as the second leading cause of accidental injury deaths globally [1]. The impact of this phenomenon is disproportionately severe in paediatric populations within school environments, where dynamic physical activity is integral to development. Beyond the immediate biomechanical trauma—ranging from wrist fractures to traumatic brain injuries (TBIs)—repeated falls in children often precipitate a psychological “fear of falling.” This acquired anxiety can lead to activity avoidance, reduced classroom participation, and long-term deconditioning, creating a vicious cycle of motor incompetence and increased injury risk [2]. Consequently, the systematic analysis and mitigation of fall risk in schools is not merely a safety protocol but an educational priority.

Historically, fall prevention strategies have relied on passive environmental modifications, such as compliant flooring or increased teacher supervision. While necessary, these measures fail to address the multifactorial aetiology of falls, which involves complex interactions between a child’s biomechanical deficits and environmental hazards. In response, the *Safe Fall—Safe Schools* initiative proposes a paradigm shift towards proactive motor competence. Leveraging the principles of Judo’s *Ukemi* (breakfall) techniques, this pedagogical programme trains children to convert vertical impact forces into horizontal rotational energy [3]. By automatizing protective responses—such as neck flexion (tucking the chin) and arm positioning—children become active agents in mitigating their own injury risk [4].

However, quantifying the acquisition of these complex motor skills in ecological school settings remains a significant technological bottleneck. Current validation relies on the *INFOSECA* observation scale, a clinical instrument that evaluates five biomechanical criteria (e.g., head protection, trunk rolling) [2]. While clinically valid, manual observation is subjective, labour-intensive, and unscalable for large student populations. This necessitates an automated, computer vision-based solution capable of “Fall Quality Analysis” distinguishing between a hazardous collapse and a controlled, protective fall.

Developing such a system presents unique computer vision challenges that differ fundamentally from geriatric fall detection (which typically flags inactivity after a fall). The biomechanics of a safe fall—specifically the “tuck-and-roll” manoeuvre—introduce severe **self-occlusions**, where the limbs obscure the torso and head from the camera’s view. Standard pose estimation frameworks, such as OpenPose or MediaPipe, frequently suffer from tracking degradation in these high-velocity, non-linear scenarios, resulting in lost keypoints and identity switching [5,6].

To bridge this gap, we present the **Safe Fall Framework**, a novel integration of state-of-the-art vision models designed for robust biomechanical assessment in the wild. We combine **YOLOv8** for real-time detection [7] with the recently introduced **Segment Anything Model 2 (SAM 2)** [8]. Unlike traditional bounding-box approaches, SAM 2 generates pixel-level segmentation masks that persist through complex occlusions, enabling our pipeline to maintain tracking continuity during rapid rolling motions.

The remainder of this paper is organised as follows. Section 2 presents the methodology of the proposed system, including data collection, vision-based modelling, and predictive analysis. Section 3 reports the main findings, comparing model performance and highlighting their implications, and discusses the integration of technological and educational perspectives. Finally, Section 4 concludes this paper and outlines directions for future research.

## 2. Materials and Methods

### 2.1. Participants and Data Collection

The study sample comprised **285 school-aged children** (ages 6–12, mean age = 9.4±1.2 years) recruited from five primary schools participating in the *Safe Fall* pedagogical programme [2]. The experimental design followed a quasi-experimental pre-test–post-test structure to evaluate the acquisition of protective motor skills. Participants were assessed at two distinct time points: a baseline pre-intervention phase (n=285) to capture spontaneous fall reactions, and a post-intervention phase (n=130) recorded after the completion of a six-week instructional unit based on Judo *Ukemi* (breakfall) techniques. Exclusion criteria included any pre-existing orthopedic injuries or neurological conditions that would contraindicate physical impact exercises.

The sample attrition (dropout) observed between the pre-test and post-test stages is a common characteristic of longitudinal studies in ecological school environments. In this study, the reduction was primarily driven by strict inclusion criteria: to accurately measure the intervention’s true pedagogical effect, students were only included in the post-test analysis if they had attended a minimum of 80% of the instructional sessions. Consequently, ordinary school dynamics—such as absences due to minor illnesses, scheduling conflicts, or non-attendance on the specific day of the post-test recording—accounted for the dropout. To robustly handle this inherent class imbalance during evaluation, non-parametric statistical tests and stratified cross-validation strategies were systematically employed in the subsequent analyses.

To ensure high *ecological validity*, data collection was conducted in standard school gymnasiums rather than controlled laboratory settings. This approach preserved the natural variability of children’s movements, addressing a common limitation in fall detection datasets, which often rely on simulated falls in sterile environments [9]. Furthermore, unlike datasets such as SisFall [10] that rely on invasive wearable sensors (IMUs), which may alter motor behaviour, we employed a non-intrusive computer vision approach.

**Figure 1:**
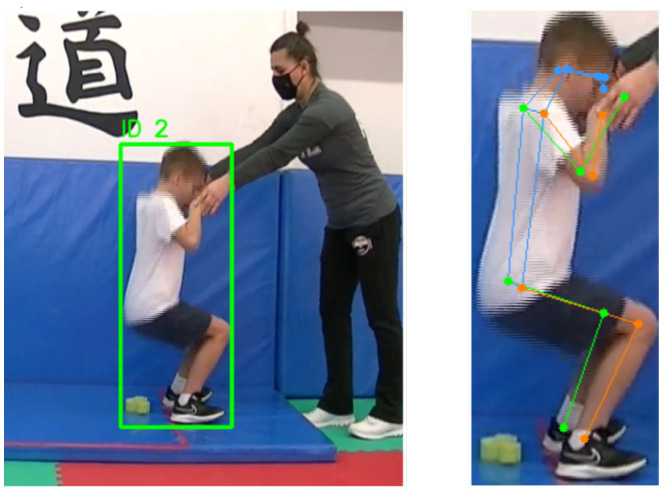
Visualisation of pose detection for an individual experiencing a fall. The left image shows the full frame with the bounding box of the individual (ID 2), while the right image details the estimated pose and the keypoints detected for the same individual during the fall.

Video data were captured using fixed RGB cameras recording at 60 fps with a resolution of 1920×1080 pixels. Cameras were positioned at a standardised height of 1.10 m (approximate chest height relative to the subjects) and oriented frontally. This viewpoint was specifically selected to maximise the visibility of critical anatomical landmarks (head, hips, knees) and minimise self-occlusion during the rotational phase of backward rolling falls (*Ushiro-Ukemi*) (see Figure 1), a challenge noted in recent pose estimation benchmarks [6].

Safety was paramount; all falls were performed on high-density polyurethane foam mats ($20\;{\mathrm{kg}/m}^{3}$) under the direct supervision of physical education specialists [3]. The recording sessions included both individual drills and paired exercises to reflect the dynamic nature of physical education classes, introducing realistic challenges such as background clutter and partial occlusions that the proposed vision pipeline is designed to resolve.

### 2.2. Vision-Based Analysis Pipeline

**Figure 2:**
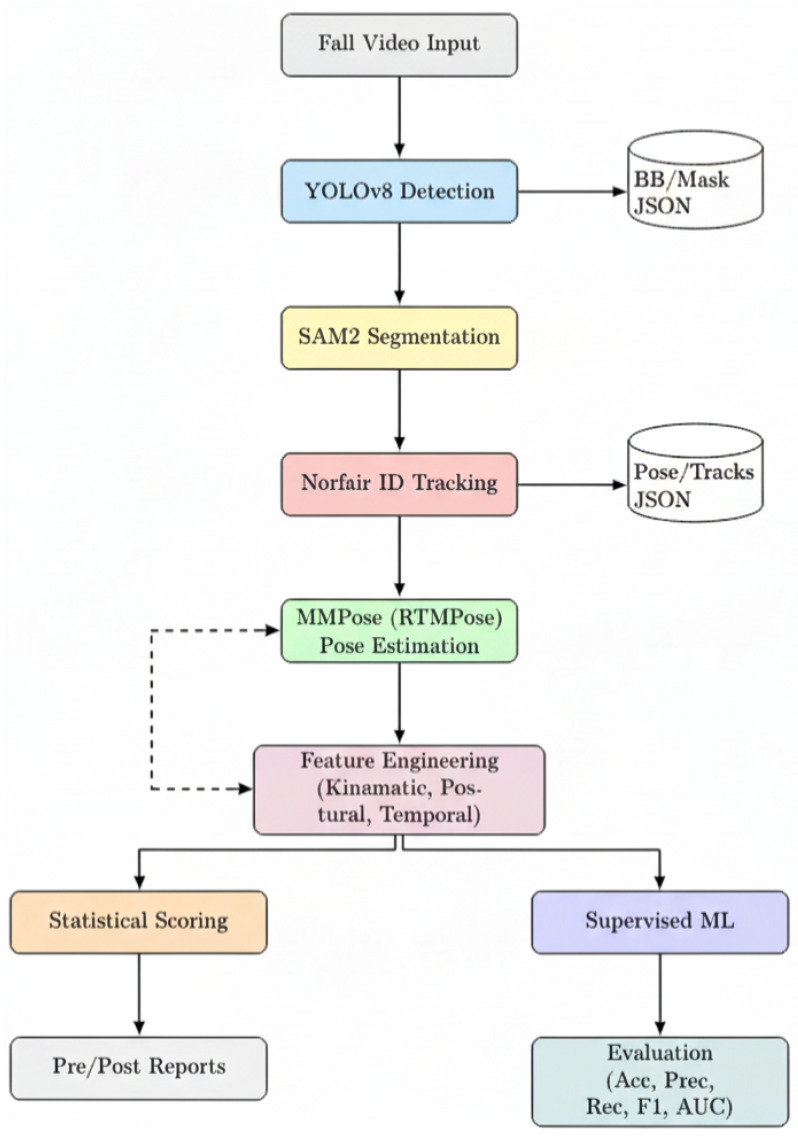
End-to-end pipeline: sequential perception (detection, segmentation, tracking), pose estimation, feature engineering, and bifurcation into statistical scoring and supervised ML, with outputs for reporting and evaluation.

To quantify fall biomechanics, we developed a modular computer vision pipeline (Figure 2) that processes raw RGB footage into structured kinematic data. The architecture follows a top-down approach, prioritizing high-precision instance segmentation to mitigate the background clutter typical of school gymnasiums. The pipeline consists of four sequential stages:

1. **Person Detection (YOLOv8):** Human instances are initially localised using **YOLOv8** [7]. We selected this single-stage detector for its optimal trade-off between inference speed and recall rates in dynamic environments. The model generates bounding box priors ($B_{t}$) for every subject in frame *t*, filtering out non-human objects (e.g., mats, equipment) with a confidence threshold of 0.5.
2. **Occlusion-Robust Segmentation (SAM 2):** A critical challenge in analysing rolling falls (*Ukemi*) is the separation of the subject from the floor mats during impact. To address this, we integrated the **Segment Anything Model 2 (SAM 2)** [8]. Unlike traditional bounding boxes, SAM 2 utilises the priors from YOLOv8 to generate pixel-perfect binary masks. This step effectively removes background noise and isolates limb contours even during complex self-occlusions, a capability lacking in older pipelines based solely on OpenPose.
3. **Multi-Object Tracking (Norfair):** To reconstruct consistent temporal trajectories, we employed **Norfair** [11], a lightweight tracking library based on Kalman filters. Norfair associates detections across frames by predicting the future position of each centroid based on its velocity history. This ensures that the identity of a child is preserved ($ID_{i}$) throughout the fall sequence, even if detection is momentarily lost during rapid rotation.
4. **Pose Estimation (RTMPose):** Finally, 2D skeletal kinematics were extracted using the **MMPose** framework. We utilised the **RTMPose** architecture (ResNet backbone) pre-trained on the COCO dataset [12]. For each tracked instance, the model regressed 17 anatomical keypoints. The output was a coordinate vector $P_{i,t}={\left\{\left(x_{k},y_{k},c_{k}\right)\right\}}_{k=1}^{17}$, where $c_{k}$ represents the confidence score for joint *k*.

The complete pipeline was optimised for reproducibility and deployed on a workstation with GPU acceleration. All processing stages were implemented in Python 3.11 using PyTorch v.2.10.cu130-based frameworks, ensuring full reproducibility of the experimental pipeline. Processing scripts generated structured outputs that included bounding boxes, masks, and skeletons for each identity in JSON format, together with synchronised video overlays for visual inspection.

### 2.3. Feature Extraction and Scoring System

To quantify the biomechanical quality of the falls, we transformed the raw skeletal coordinates $P_{i,t}$ into a set of 15 interpretable descriptors. Features were extracted frame-by-frame and aggregated across the temporal window of the fall event to capture the dynamics of the *Ukemi* technique.

#### 2.3.1. Kinematic Features (Impact Dynamics)

Velocity and acceleration of critical body segments are direct indicators of impact severity. We focused on the hip centre (approximating the Centre of Mass, CoM) and the head [13]. The instantaneous vertical velocity $v_{y}$ at frame *t* was computed as

(1) $$v_{y}\left(t\right)=\frac{y_{hip}\left(t\right)-y_{hip}\left(t-\Delta t\right)}{\Delta t}\cdot fps$$

where fps is the recording frame rate. We extracted the **Peak Impact Velocity ($v_{max}$)** and the **Head Descent Trajectory**. A controlled, safe fall was characterised by a gradual deceleration curve, whereas a hazardous fall exhibited a sharp spike in $v_{max}$ at ground contact [4].

#### 2.3.2. Postural and Geometric Metrics

To evaluate the “tuck-and-roll” strategy, we calculated relative joint angles and bounding box transformations:

- **Trunk Inclination ($\theta_{trunk}$):** Defined as the angle between the vertical axis and the vector connecting the mid-hip to the mid-shoulder. A rapid transition from $0^{\circ }$ (vertical) to $90^{\circ }$ (horizontal) served as a primary fall trigger [6].
- **Bounding Box Aspect Ratio (AR):** Calculated as $AR=\frac{Width_{bbox}}{Height_{bbox}}$. An AR>1.0 typically indicated a transition to a lying state, distinguishing falls from daily activities like sitting [14].
- **Neck Protection Index:** We measured the Euclidean distance between the chin keypoint and the sternum. A minimised distance indicated active neck flexion (chin-to-chest), a critical safety criteria in the INFOSECA scale to prevent occipital trauma.

#### 2.3.3. Temporal Segmentation

Automatic segmentation of the fall phases (Pre-fall, Impact, Post-fall) was achieved by analysing the zero-crossing rate of the vertical velocity. The **Impact Phase** was defined as the temporal window ±200 ms around the maximum deceleration peak of the hips.

#### 2.3.4. The Safe Fall Composite Score ($S_{safe}$)

The fifteen descriptors described above were used for the statistical analyses and clustering procedures, as they provided interpretable biomechanical indicators of fall execution. For the supervised learning stage, however, a higher-dimensional representation was derived from the temporal evolution of the skeletal coordinates, including frame-level kinematic descriptors and temporal derivatives. This process resulted in a total of 313 input features used for predictive modelling.

To synthesise these variables into a single evaluative metric, we developed a hybrid composite score combining hypothesis-driven and data-driven criteria. First, Cohen’s d (statistical effect size from the pre/post-intervention comparison) was utilised as a filter to select only the biomechanically significant features. Second, the actual weight coefficients ($w_{j}$) for these normalised features ($f_{j}$) were determined by their discriminative power (Mean Decrease in Impurity) extracted from the Random Forest classifier.

(2) $$S_{safe}=\sum_{j=1}^{N}w_{j}\cdot f_{j}$$

This hybrid strategy ensured that the final scoring rule was both biomechanically relevant (hypothesis-driven) and optimised for predictive accuracy (data-driven).

**Label Generation and Inter-Rater Reliability:** To operationalise the clinical INFOSECA scale into binary labels (“Safe Fall” vs. “Hazardous Fall”) for the supervised machine learning experiments, a strict protocol was established. The INFOSECA scale evaluates five biomechanical items. A sequence was labeled as a “Safe Fall” if and only if the most critical protective responses were successfully executed: active neck flexion (chin-to-chest to prevent head trauma) and controlled trunk rolling, coupled with the absence of hazardous reactions such as extending straight arms towards the ground (FOOSH). The annotation was performed by two independent experts in sports biomechanics. The inter-rater agreement was high, yielding a Cohen’s Kappa of 0.89. Any ambiguous cases (representing approximately 4.5% of the dataset) were reviewed and resolved through consensus with a third senior researcher, ensuring a transparent and reproducible ground truth.

### 2.4. Machine Learning Classification Framework

To automate the assessment of fall quality, we formulated a supervised binary classification task distinguishing between *Safe Falls* (correct Ukemi execution) and *Hazardous Falls* (uncontrolled collapse). Leveraging the extracted feature vectors, we implemented and compared both ensemble-based and deep learning architectures to capture linear and non-linear biomechanical dependencies.

#### 2.4.1. Ensemble Learning (Random Forest)

We prioritised **Random Forest (RF)** [15] as our primary baseline due to its high interpretability and resistance to overfitting in high-dimensional feature spaces. Recent comparative studies on fall datasets indicate that RF classifiers often outperform other traditional methods (e.g., SVM, k-NN) by effectively handling the non-linear boundaries of kinematic data [16]. Crucially, RF allows for **Feature Importance Analysis** (via Gini impurity decrease), enabling us to quantify which biomechanical variables (e.g., hip velocity vs. neck angle) contribute most to a safe landing. This “White-Box” approach is essential for validating the pedagogical effectiveness of the Safe Fall programme [14].

#### 2.4.2. Deep Sequence Modeling (LSTM and ST-GCN)

To capture the temporal evolution of the fall trajectory, we employed deep neural architectures:

- **Recurrent Networks (LSTM):** We utilised Long Short-Term Memory (LSTM) networks [17] to process the sequential inputs of kinematic features. Unlike static classifiers, LSTMs maintain an internal memory state, allowing the model to distinguish between a “controlled descent” and a “sudden drop” based on the velocity history of the movement [18].
- **Spatio-Temporal Graph Convolution (ST-GCN):** Given that human data is inherently structured as a graph (joints connected by bones), we also explored Spatial–Temporal Graph Convolutional Networks. This architecture exploits the natural topology of the skeleton extracted by MMPose, learning spatial dependencies (e.g., knee-hip coordination) and temporal dynamics simultaneously, a method that has shown state-of-the-art performance in recent skeleton-based fall detection benchmarks [19].

#### 2.4.3. Evaluation Protocol

To ensure the robustness of the models and prevent data leakage—a common pitfall where models memorise subject-specific features (e.g., clothing)—we implemented a **Leave-One-Subject-Out (LOSO)** cross-validation strategy. Model performance was assessed using Accuracy, Precision, Recall, F1-Score, and the Area Under the Receiver Operating Characteristic Curve (ROC-AUC). Special emphasis was placed on **Sensitivity (Recall)**, as minimizing False Negatives (labeling a dangerous fall as safe) is critical in injury prevention scenarios [14].

## 3. Results and Discussion

This section evaluates the Safe Fall framework across three complementary dimensions. First, we quantified the pedagogical impact of the intervention by comparing pre- and post-test kinematic metrics (n=415). Second, we applied unsupervised clustering to identify latent motor profiles and their shift towards protective strategies. Finally, we validated the automated vision pipeline by assessing the classification performance of the Random Forest and deep learning models in distinguishing safe from hazardous falls.

### 3.1. Comparative Metrics Analysis (Pre-Test vs. Post-Test)

To quantify the biomechanical impact of the *Safe Fall* programme, we compared the motor response to unexpected **backward falls** between the pre-test (n=285) and post-test (n=130) cohorts. We utilised a multidimensional framework of 15 metrics categorised into temporal indicators, kinematic parameters (body orientation, CoM displacement), stability measures, and protective strategies. Given that Shapiro-Wilk tests indicated violations of normality, we applied the non-parametric Mann–Whitney *U* test. Overall, **9 out of 15 metrics (60%)** showed statistically significant differences (p<0.05), providing objective evidence of a shift from uncontrolled dorsal impacts to managed backward rolling strategies (UshiroUkemi).

**Kinematic Smoothness and Impact Mitigation.** The intervention successfully modified the internal structure of the backward fall (Table 1). While median values for velocity showed a slight increase, the *mean* values decreased significantly (p<0.001), indicating a reduction in movement skewness and smoother kinematics. Biomechanically, this suggests that trained children exhibit superior neuromuscular control during the retropulsion phase, regulating their descent rather than succumbing passively to gravity [4].

**Spatial Expansion and Rolling Mechanics.** A critical indicator of the safe backward fall is the expansion of the movement footprint. There were highly significant increases in **Movement Range X** ($p<10^{-4}$) and **Movement Range Y** (p=0.0007). In the context of a backward fall, the increase in the horizontal range (X) confirms that post-intervention subjects utilised the mat’s surface to execute a **backward roll**. This mechanism converts potentially injurious vertical energy into horizontal rotational motion, protecting the spine and head from direct impact [2].

**Decisive Descent and Commitment.** A pivotal finding was the marked rise in **Avg. Descent Rate** (+61.4%, $p<10^{-6}$) and **Total Vertical Change** (p=0.0007). This demonstrates that trained children commit to the protective technique decisively—typically by executing a rapid squat to lower the Center of Mass (CoM) before rolling—rather than hesitating or braking late with stiff limbs, a hazardous behaviour observed in the pre-test group associated with wrist fractures (FOOSH) [4].

**System Robustness and Statistical Validation.** Peak magnitude metrics such as **Max. Velocity** (p=0.062) did not reach significance, suggesting that peak physical bursts are governed by gravity rather than skill. Crucially, the **Avg. Confidence** score of the vision system saturated at 1.0 in both groups. This validates the robustness of our **YOLOv8 + SAM 2** pipeline, demonstrating that tracking remains reliable even during the severe self-occlusions typical of a backward roll (where legs obscure the torso) [6]. All findings held under False Discovery Rate (FDR) corrections, confirming that the improvements were not artifacts of the unequal sample sizes.

**Interpretation of Non-Significant and Saturated Metrics.** Not all metrics exhibited statistically significant changes, yet these null results provide crucial context for understanding the biomechanics of the backward fall (Table 2). Peak-based metrics, such as **Max. Velocity** (p=0.062) and **Max. Acceleration** (p=0.099), showed positive trends but did not reach significance. This suggests that while the intervention improved the *average* control of the descent (smoother movement), the peak physical bursts were largely governed by the laws of physics (gravity acting on the falling body) and individual anthropometry, which remained constant [4].

Similarly, the lack of change in coarse activity counters, such as **High-Movement Frames** (p=0.910) and **Movement Intensity** (p=0.908), indicates that the quantity of motion remained similar. The improvement stems from *how* children organised their kinematics (quality) rather than simply “moving more” or “moving less”. A panicked flailing of limbs (pre-test) generated as much pixel variation as a controlled backward roll (post-test), but the latter is protective while the former is hazardous. Finally, the **Avg. Confidence** score saturated at 1.0 in both groups. This ceiling effect confirms that our vision pipeline (YOLOv8 + SAM 2) is robust enough to track subjects perfectly in both chaotic and controlled scenarios, validating the tool for real-world usage [6].

**Overall Effectiveness of the Intervention.** Synthesizing the results across all domains, the *Safe Fall* programme demonstrated high pedagogical effectiveness. A total of 15 metrics were analysed, of which **9 (60%)** showed statistically significant pre–post differences (Table 3). Six of these were highly significant (p<0.001), reflecting systematic improvements in movement control.

The pattern of improvement was distinct across categories: 100% of the **Fall-specific metrics** (Descent Rates, Vertical Change) improved, indicating that children learned to commit to the backward fall strategy (squatting and rolling) rather than resisting it. Similarly, 66.7% of **Stability** and 55.6% of **Movement** metrics improved, evidencing a shift towards smoother, more spatially distributed kinematics. This multidimensional validation confirms that the intervention successfully overwrote the natural “stiffening” reflex with a trained, energy-dissipating motor habit [3].

**Interpretation of Non-Significant Metrics.** Five metrics did not reach statistical significance: **Max. Velocity** (p=0.062), **Max. Acceleration** (p=0.099), **Position Variability Y**, **High-Movement Frames**, and **Movement Intensity**. These measures either remained highly variable across individuals or saturated at floor/ceiling values, offering limited discriminative power. Crucially, the lack of change in coarse activity counters suggests that the intervention primarily influenced the *organisation and quality* of the movement rather than its overall magnitude. In other words, trained children do not necessarily move “less” or “slower” at peak moments, but they organise their kinetic chain differently to protect vital areas [4].

**Magnitude of Effect and Key Findings.** Beyond statistical significance, the practical impact of the intervention was substantial. Large effect sizes (Cohen’s d>0.8) were observed in multiple metrics, including Avg. Velocity, Avg. Acceleration, and Total Vertical Change. The most notable percentage changes were found in **Movement Range X** (+21.4%), **Position Variability X** (+22.5%), and **Avg. Descent Rate** (+61.4%). These dramatic increases reflect the acquisition of a decisive and coordinated protective strategy: children learned to expand their body horizontally (rolling) and commit to the descent rapidly (squatting) to dissipate energy, rather than freezing in an upright position [2].

**Recommendations and Methodological Considerations.** The results confirm the effectiveness of the *Safe Fall* programme and support its continued implementation in physical education curricula. The current protocol appears robust, with particular success in sustaining improvements in velocity control and spatial ranges. From a methodological standpoint, the inclusion of effect size measures validates the practical significance of the observed improvements beyond *p*-values. Future work should consider corrections for multiple comparisons and investigate why certain peak-based metrics remain insensitive, potentially exploring higher frame-rate capture or 3D depth sensors to capture finer rotational details [6].

### 3.2. Clustering Analysis: Identification of Latent Motor Profiles

**Figure 3:**
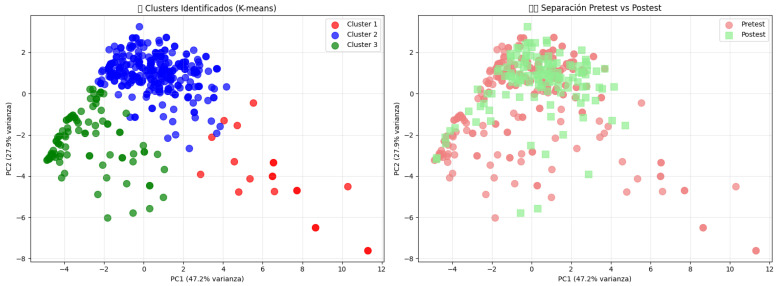
**Latent motor profiles identified via K-means clustering.** The scatter plot visualises the distribution of participants along the first two principal components. **Cluster 1 (Red)** represents high-intensity/erratic falls typical of untrained panic; **Cluster 3 (Green)** represents passive/freezing responses linked to fear; **Cluster 2 (Blue)** represents the desired controlled, spatially distributed protective strategy (*Ukemi*). Note the shift of post-test participants converging towards Cluster 2.

To explore the heterogeneity in fall responses beyond average metrics, we conducted an unsupervised clustering analysis using the *k*-means algorithm on the 15 extracted kinematic features (n=415). Unlike traditional binary classifications (Fall vs. ADL) found in public datasets like SisFall or MobiAct [10], which focus solely on detection, this approach reveals the *qualitative spectrum* of the motor response. Dimensionality reduction via Principal Component Analysis (PCA) revealed a clear separation of motor strategies along the first two components (PC1: 47.2% variance, PC2: 27.9% variance), identifying three distinct motor profiles (Figure 3).

**Cluster 1: High-Intensity Hazardous Reactions (n=20).** Predominantly composed of pre-test subjects (17/20), this cluster represented a “chaotic” response pattern characterised by substantially elevated **Movement Intensity** (+831.5% above sample mean) and excessive high-movement frames. This profile aligns with the “uncontrolled descent” patterns frequently observed in the unfiltered raw data of public datasets, where untrained subjects exhibit sharp acceleration spikes and erratic limb flailing [10]. Biomechanically, this suggests a panic response, where high kinetic energy is generated but not dissipated efficiently, drastically increasing the risk of impact injury compared to controlled strategies.

**Cluster 2: Controlled Protective Strategy (n=300).** Containing the majority of post-test participants, this cluster represents the target biomechanical profile. While showing lower movement intensity than Cluster 1 (−67.2%), subjects exhibited superior **spatial control** and extended horizontal movement range. Unlike the rigid falls seen in Cluster 3, this profile corresponded to the efficient execution of the *Ukemi* technique: transforming vertical impact energy into rotational motion. This confirms the efficacy of the intervention in overwriting natural reflexes with the “tuck-and-roll” strategies advocated in the martial arts biomechanics literature [2].

**Cluster 3: Passive/Freezing Response (n=95).** This group was defined by low-magnitude, underactive responses, featuring significantly reduced descent rates and minimal position variability. This kinematic signature mirrors the “stiffening” or “freezing” strategy often reported in geriatric falls associated with the *fear of falling* [4]. Although the low velocity might seemingly imply safety, the lack of spatial expansion indicates that the subject absorbs the impact directly through skeletal structures (e.g., hip or spine) rather than dissipating it through movement.

**Pedagogical Impact and Distribution Shift.** A Chi-square test of independence confirmed a significant association between the intervention phase and cluster membership ($\chi^{2}=11.19$, p=0.0037). Post-test participants were significantly over-represented in **Cluster 2 (Controlled Strategy)**, while pre-test participants predominated in the hazardous extremes of Cluster 1 (Chaotic) and Cluster 3 (Passive). This shift demonstrates that the *Safe Fall* programme effectively modifies the natural motor response: it dampens the dangerous erratic behaviour of Cluster 1 and mobilises the freezing behaviour of Cluster 3, converging diverse students towards a unified, safer motor standard.

### 3.3. Predictive Analysis: Biomechanical Markers of Skill Acquisition

To identify the specific biomechanical signatures that distinguish trained from untrained participants, we formulated a supervised classification task comparing pre-test and post-test samples. Although related, these two tasks are conceptually distinct: the intervention classification (pre-test vs. post-test) captures skill acquisition, while the fall quality classification evaluates the biomechanical safety of individual fall executions. Unlike binary fall detection systems that focus solely on identifying the occurrence of a fall event [14], this analysis aims to quantify the *learning effect* embedded in the movement patterns.

We trained a Random Forest classifier ($n_{estimators}=100$, Gini criterion) on the 15 extracted kinematic features. Random Forest was selected due to its strong performance in handling tabular kinematic data and its high interpretability compared to more opaque models, as reported in previous sensor-based classification studies [16,20]. To address the class imbalance (pre n=285, post n=130), we applied a stratified 5-fold cross-validation scheme.

**Classification Performance.** The model achieved a mean accuracy of **77.1% ± 2.4%** and an Area Under the Curve (AUC) of 0.81. While lower than the performance obtained in the fall-quality classification task, this result is notable for a skill assessment task. It indicates that the intervention induces a measurable and consistent change in the biomechanical “fingerprint” of the fall, allowing the algorithm to correctly identify a trained student in nearly four out of five cases solely based on their movement kinematics.

**Feature Importance and Biomechanical Interpretation.** The Random Forest analysis provided an interpretability ranking based on the mean decrease in impurity (Table 4). The results reveal a hierarchy of motor skills:

1. **Decisive Entry (Descent Rate):** The **Avg. Descent Rate** emerged as the single most predictive feature (relative importance: 0.2042). Biomechanically, this suggests that hesitation and stiffness observed in the pre-test group are associated with a lower descent rate. In contrast, trained children execute a more decisive lowering of the Center of Mass (CoM)—typically through a squatting motion—to initiate the *Ukemi* roll [3].
2. **Spatial Redistribution (Variability and Range):** Features describing spatial occupancy (**Position Variability Y**, **Movement Range X**) ranked highly. This corroborates the clustering results: trained falls were not static vertical drops but dynamic events where energy was redistributed horizontally across the mat. High variability in the vertical axis (Y) suggests active modulation of height during the squat-to-roll transition [4].
3. **Controlled Dynamics vs. Peak Bursts:** While **Avg. Velocity** and **Avg. Acceleration** were predictive, peak values (Max. Velocity/Acceleration) were less dominant. This indicates that skill acquisition is better reflected in the consistency and smoothness of the trajectory rather than in suppressing peak forces governed by gravity [13].

**Table 4:** **Biomechanical determinants of intervention status.** Top ten predictive features distinguishing pre-test (untrained) and post-test (trained) participants, ranked by Random Forest feature importance.

Biomechanical Feature	Relative Importance	Physical Interpretation
**1. Avg. Descent Rate**	**0.2042**	Speed of CoM lowering (Decisiveness)
**2. Position Variability Y**	0.0917	Vertical control (Squatting/Rolling)
**3. Avg. Velocity**	0.0907	Overall movement smoothness
**4. Movement Range X**	0.0886	Horizontal energy dissipation (Rolling)
5. Max. Velocity	0.0768	Peak kinetic energy
6. Position Variability X	0.0730	Lateral spatial usage
7. Max. Acceleration	0.0728	Impact severity peak
8. Avg. Acceleration	0.0625	Descent control
9. Total Vertical Change	0.0602	Completeness of fall technique
10. Max. Descent Rate	0.0561	Peak downward velocity

These findings provide data-driven validation of the *Safe Fall* pedagogical approach: the intervention successfully overwrites the natural instinct to stiffen and resist gravity with a trained motor program characterised by decisive entry and spatial expansion [2].

### 3.4. Predictive Modelling: Supervised Classification of Fall Quality

To automate the assessment of fall quality, supervised classification models were trained on the comprehensive feature set extracted via MMPose. At the subject level, the dataset contained 415 fall observations (pre-test and post-test combined). However, for the machine learning models, the data were analysed at the frame level in order to capture the temporal dynamics of the movement. After segmentation of the fall sequences, this resulted in a dataset of 49,192 frame-level samples represented by 313 kinematic input features used for training and evaluation. Each fall sequence was segmented into temporal windows corresponding to the fall event, and features were computed at the frame level to capture the fine-grained kinematic evolution of the movement. Given the inherent class imbalance (pre-test n= 41,223 vs. post-test n= 7969), representative of real-world scenarios where “correct” falls are less frequent than untrained ones, we prioritised metrics robust to skewness, specifically the F1-Score and the Area Under the Receiver Operating Characteristic Curve (AUC) [14].

To evaluate the model’s performance while rigorously preventing data leakage, we utilised a strict **Subject-Independent 5-Fold Cross-Validation (GroupKFold)**. Although the machine learning models operated on the 49,192 frame-level samples to capture temporal dynamics, the cross-validation split was enforced strictly at the subject level. Consequently, all frames belonging to a specific participant were assigned exclusively to either the training fold or the testing fold. This subject-independent protocol guarantees that the reported metrics reflect the model’s true capability to generalise to unseen individuals, rather than memorizing subject-specific visual or kinematic artifacts.

Table 5 details the subject-independent 5-fold cross-validation results. The **Random Forest (RF)** classifier emerged as the superior architecture, achieving an accuracy of **98.3%**, an F1-Score of 0.983, and a near-perfect AUC of 0.998. This performance aligns with recent findings in sensor-based fall detection, where ensemble methods often outperform single classifiers due to their ability to model non-linear biomechanical boundaries without overfitting [16,20].

The dominance of ensemble methods (Random Forest and Gradient Boosting) over the Support Vector Machine (SVM) is notable. While SVMs are effective in high-dimensional spaces, they showed sensitivity to the dataset’s imbalance, yielding a significantly lower F1-score (0.766). In contrast, the Random Forest model successfully captured the complex decision boundaries of “safe falling” (e.g., specific combinations of knee flexion and neck protection) while maintaining computational efficiency suitable for edge deployment [8]. The Multilayer Perceptron (MLP) provided a competitive neural baseline (93.2%), but the slight performance gap suggests that for structured tabular kinematic data, the hierarchical decision capability of trees is more sample-efficient than fully connected layers [17].

Our proposed framework compares favourably with recent vision-based benchmarks. For instance, Wang et al. (2024) reported an accuracy of 89.99% using Random Forest with BlazePose features [14], and Kong et al. (2025) achieved an F1-score of 91.4% using an SVM on geometric features [20]. The superior performance of our model (98.3%) can be attributed to the high fidelity of the MMPose-extracted skeleton and the comprehensive feature engineering that captures dynamic velocities and angular relations, rather than just static poses.

## 4. Conclusions

This study presented a framework for the automated assessment of safe falling strategies using machine vision and predictive modelling. By successfully bridging biomechanics, pedagogy, and artificial intelligence, the results show that the proposed approach is capable of capturing meaningful changes in fall execution after a judo-inspired *Ukemi* intervention, while also providing an objective, scalable basis for evaluating fall quality in ecological school settings.

As a primary quantitative outcome, the pre–post analysis showed measurable changes in several kinematic variables, with approximately **60%** of the analysed biomechanical metrics presenting statistically significant differences. In particular, the observed changes in average velocity, acceleration, and spatial displacement ranges suggest that the intervention promoted more controlled and better distributed movement patterns during the fall. These kinematic shifts reflect a transition towards an “active collapse” strategy. These changes are consistent with a safer execution of the movement, in which impact energy is managed through improved body control and dissipated through spatial redistribution, rather than absorbed directly by the skeletal structure.

In addition to these physical improvements, this pattern of active energy dissipation was further supported by the protective strategy scores and clustering analysis, which provided crucial insights into the psychological dimension of falling. After the intervention, participants more frequently exhibited coordinated protective responses and were more often grouped within the profile associated with controlled falling behaviour, while the proportion of passive or freezing responses decreased. Because joint stiffening and hesitation are physical manifestations of the fear of falling, this shift confirms that the programme successfully equips children with the motor confidence to yield to gravity. Taken together, these findings indicate that the programme contributed to the acquisition of safer motor strategies during backward falls.

To complement the biomechanical and psychological findings, from a technological perspective, the predictive analysis confirmed that pose-derived features provide sufficient information to distinguish between different levels of fall quality, moving beyond simple binary fall detection to provide qualitative motor assessment. The Random Forest model achieved the best performance, reaching **98.3% accuracy** and an **AUC of 0.998**. The most informative variables were mainly related to descent dynamics and spatial redistribution, reinforcing the relevance of these factors for the assessment of fall quality. An additional limitation of the current study is the restricted age range of the participants (school-aged children, 6–12 years old). While our within-subject pre/post design internally controlled for baseline biomechanical and anthropometric differences (e.g., height, weight) among the children, the suitability of the proposed vision-based system for other age categories was not assessed. Fall kinematics vary significantly across the lifespan; for instance, the elderly population exhibits different centre-of-mass dynamics, slower reflex times, and distinct injury mechanisms (such as lateral hip impacts leading to fractures) compared to pediatric populations [13,14]. Although our engineered features—such as relative descent rate and spatial expansion—were relatively robust to individual size variations within our sample, generalizing this framework to adults or geriatric populations would require further validation and potentially the integration of age-specific kinematic thresholds. Future research should evaluate the framework’s adaptability across diverse demographic cohorts to establish a universal fall quality assessment tool.

Despite these promising results, some limitations remain that must be addressed in future research. While our pipeline successfully managed gross self-occlusions during rolling using advanced segmentation, certain fine-grained biomechanical variables, particularly those associated with head protection (e.g., exact cervical angles) and complex rolling phases, are still difficult to estimate reliably from 2D pose data because of severe self-occlusion. Future work should therefore explore multi-camera configurations, 3D reconstruction strategies, or the integration of lightweight inertial sensing in order to improve the analysis of complex fall mechanics. Additionally, deploying these models on Edge AI devices will be essential to provide real-time, on-site feedback for physical educators.

Overall, the proposed framework supports the use of vision-based motion analysis and machine learning as an objective and scalable tool for assessing safe falling strategies in educational and sports-related contexts, ultimately contributing to injury reduction, mitigation of fear, and lifelong motor safety.

Furthermore, the quasi-experimental design and the lack of a randomised control group warrant a cautious interpretation of the pedagogical effectiveness. While the pre-test to post-test improvements were strongly associated with the *Safe Fall* intervention, potential confounders—such as natural motor maturation over the six-week period or increased familiarity with the testing environment—cannot be definitively ruled out. Future research should employ Randomised Controlled Trials (RCTs) with paired analyses to establish stronger causal inferences regarding intervention effects.

## Figures and Tables

**Table 1:** Significant pre–post differences in kinematic metrics during backward falls. Medians (Md) are reported for robustness; arrows denote direction of change.

Metric	Pre Md	Post Md	Change	*p*
**Avg. Velocity**	5.17	6.71	↑ slight (mean ↓)	<0.001 ***
**Avg. Acceleration**	6.00	7.87	↑ (mean ↓)	0.0046**
**Movement Range X**	209.83	233.93	↑ (rolling)	<10^−4^ ***
**Movement Range Y**	224.60	244.63	↑	0.0007***
**Position Variability X**	67.63	77.52	↑	0.0003***
**Velocity Variability**	14.19	17.76	↑	0.0169 *
**Max. Descent Rate**	71.49	96.96	↑	0.0046 **
**Avg. Descent Rate**	0.84	1.52	↑	<10^−6^ ***
**Total Vertical Change**	224.60	244.63	↑	0.0007 ***

*Notes.* Pre-test n=285, Post-test n=130. Mann–Whitney *U* used where assumptions were violated. Increases in Movement Range X indicate effective backward rolling distribution. Significance codes: * p<0.05, ** p<0.01, *** p<0.001. Arrow ↑ indicates an increase from pre-test to post-test; arrow ↓ indicates a decrease from pre-test to post-test.

**Table 2:** Non-significant pre–post differences and saturated measures. Peak-based metrics are less sensitive to skill acquisition than time-averaged descriptors.

Metric	Pre Md	Post Md	*p*
**Max. Velocity**	121.13	137.95	0.062
**Max. Acceleration**	141.07	190.99	0.099
**Position Variability Y**	82.07	83.59	0.477
**High-Movement Frames**	0.000	0.000	0.910
**Movement Intensity**	0.000	0.000	0.908
**Avg. Confidence**	1.000	1.000	0.98

**Table 3:** Summary of significant results by metric category, highlighting the high effectiveness in fall-specific mechanics.

Category	Total Metrics	Significant	Examples
**Movement**	9	5 (55.6%)	Avg. Velocity (↓), Range X (↑)
**Stability**	3	2 (66.7%)	Position Var. X (↑), Vel. Var. (↑)
**Fall**	3	3 (100.0%)	Descent Rate (↑), Vert. Change (↑)

*Notes.* Arrow ↑ indicates an increase from pre-test to post-test; arrow ↓ indicates a decrease from pre-test to post-test.

**Table 5:** Performance of predictive models (5-fold cross-validation). Random Forest and Gradient Boosting demonstrate superior robustness against class imbalance compared to SVM.

Model	Accuracy	Precision	Recall	F1-Score	AUC
**Random Forest**	**0.983**	**0.983**	**0.983**	**0.983**	**0.998**
Gradient Boosting	0.964	0.964	0.964	0.963	0.991
Multilayer Perceptron	0.932	0.930	0.932	0.931	0.957
Logistic Regression	0.914	0.910	0.914	0.908	0.934
Support Vector Mach.	0.839	0.865	0.839	0.766	0.871

## Data Availability

The datasets generated and analysed during the current study involve RGB video recordings of minors. Due to strict ethical privacy restrictions, the European General Data Protection Regulation (GDPR), and the Spanish Organic Law 3/2018 on Personal Data Protection and digital rights guarantees, the raw video data are not publicly available to prevent facial and biometric identification of the children. However, the anonymised tabular dataset containing the extracted skeletal kinematic features used for the predictive modelling is available from the corresponding author upon reasonable request and subject to ethical approval.

## Abbreviations

The following abbreviations are used in this manuscript:

AUC	Area Under the Curve
CoM	Centre of Mass
FDR	False Discovery Rate
FOOSH	Fall on Outstretched Hand
INFOSECA	Instrument for the Observation of Safe Falling Skills in Schoolchildren
LOSO	Leave-One-Subject-Out
LSTM	Long Short-Term Memory
MMPose	OpenMMLab Pose Estimation Framework
PCA	Principal Component Analysis
RF	Random Forest
ROC	Receiver Operating Characteristic
RTMPose	Real-Time Multi-Person Pose Estimation
SAM 2	Segment Anything Model 2
ST-GCN	Spatial–Temporal Graph Convolutional Network
TBI	Traumatic Brain Injury
WHO	World Health Organisation
